# Maternal Dietary Pattern in Pregnancy and Behavioral Outcomes at 4 Years of Age in the Piccolipiù Cohort: Potential Sex-Related Differences

**DOI:** 10.3390/nu17172814

**Published:** 2025-08-29

**Authors:** Letizia Leccese, Lorenza Nisticò, Martina Culasso, Costanza Pizzi, Vieri Lastrucci, Luigi Gagliardi, Sonia Brescianini

**Affiliations:** 1 Centre for Behavioural Science and Mental Health, Istituto Superiore di Sanità, 00161 Rome, Italy; letizia.leccese@iss.it (L.L.); sonia.brescianini@iss.it (S.B.); 2 Department of Epidemiology, ASL Roma 1, Lazio Regional Health Service, 00154 Rome, Italy; 3 Cancer Epidemiology Unit, Department of Medical Sciences, University of Turin, 10126 Torino, Italy; 4 Epidemiology Unit, Meyer Children’s Hospital IRCCS, 50139 Florence, Italy; 5 Department of Mother and Child Health, Azienda USL Toscana Nord Ovest, 56121 Pisa, Italy

**Keywords:** dietary patterns, pregnancy, maternal nutrition, child neurodevelopment, cognitive development, behavioral problems, sex-related differences, birth cohort

## Abstract

**Background**: The fetal period is critical for neurodevelopment, with maternal diet emerging as a key environmental factor influencing long-term child health. This study investigated the associations between maternal dietary patterns during pregnancy and neurocognitive and behavioral outcomes in 4-year-old children, with a particular focus on sex-related differences. **Methods:** We used data from the Piccolipiù Italian birth cohort, including 2006 mother/child pairs. Maternal dietary intake during pregnancy was assessed via a questionnaire and categorized into distinct patterns using Principal Component Analysis (PCA). Child neurodevelopment was evaluated at age 4 using the Wechsler Preschool and Primary Scale of Intelligence (WPPSI) and the Child Behavior Checklist (CBCL 1.5–5). Linear and logistic regression models were employed, adjusting for potential confounders and stratifying by child sex. **Results:** Two major maternal dietary patterns were identified: “Processed and high-fat foods” and “Fresh foods and fish”. Higher maternal adherence to the “Processed and high-fat foods” pattern was associated with increased externalizing behaviors in offspring (β = 0.88; 95%CI 0.28–1.49; *p* = 0.004). In males, this pattern was associated with an increased clinical risk of Attention Deficit Hyperactivity Disorder (ADHD) (OR (Odds Ratio) = 1.13; 95%CI: 1.02–1.26; *p* = 0.021). **Conclusions:** Our findings indicate that maternal consumption of a diet rich in processed and high-fat foods during pregnancy is associated with increased behavioral problems in children, with sex-specific vulnerabilities: slightly higher externalizing behaviors in girls and an increased risk of ADHD in boys. These results underscore the importance of promoting healthy maternal dietary patterns during pregnancy as a targeted early prevention strategy for supporting child neurodevelopment.

## 1. Introduction

The fetal period plays a vital role not only in perinatal health but also in shaping child health, lifelong well-being, and even the health of future generations [1]. It represents the most rapid phase of human cell growth and differentiation [2]. During this highly sensitive period, the fetus is particularly vulnerable to adverse in utero environmental exposures, which are influenced by maternal health, nutritional status, and broader environmental conditions such as the home, neighborhood, and society [3]. Growing evidence suggests that the human epigenome—responsible, among other factors, for regulating gene expression—is largely established during the fetal period through key processes such as DNA methylation and demethylation [4]. These changes happen at the interface between genes and the environment, providing a biological pathway through which factors like nutrition, psychosocial stress, environmental pollutants, medications, and passive exposure to cigarette smoke can impact not only fetal development but also health outcomes across the lifespan [5].

A meta-analysis on diet quality during pregnancy and cognitive and behavioral outcomes in children published in 2017 by Borge et al. [6] concluded that a better-quality diet in pregnancy had a small but significant effect on child neurodevelopment. Evidence suggests that prenatal dietary patterns can influence neurodevelopmental trajectories [7], potentially through mechanisms involving inflammation, oxidative stress (OS), and epigenetic programming [8,9,10]. Although individual nutrients such as folate, omega-3 fatty acids, and iron have been linked to neurodevelopmental outcomes [11,12], increasing attention has shifted toward examining overall dietary patterns, which better capture the complexity of dietary intake and potential synergistic effects of foods consumed together [13]. Indeed, analyzing dietary patterns can better capture the cumulative and interactive effects of the overall diet, providing a more realistic measure of nutritional exposure [14,15]. Several studies have applied this approach in maternal nutrition. For example, Ojeda-Granados et al. [13] identified both healthy (Mediterranean-like/diverse) and Western dietary patterns in women, showing that healthier patterns were richer in essential micronutrients and associated with better overall diet quality, while Western-type patterns were linked to less favorable metabolic profiles. In a large US cohort, maternal adherence to a higher-quality diet during pregnancy was associated with better visual/spatial skills in early childhood and with better intelligence and executive function in mid-childhood [7]. More recently, another study [16] reported that a Western dietary pattern in pregnancy was strongly associated with increased risk of Attention Deficit Hyperactivity Disorder (ADHD) and autism diagnoses in children at 10 years of age, particularly in males and those with higher genetic predisposition.

Diet profoundly influences the composition and metabolic activity of the gut microbiota—particularly through modulating microbial-derived metabolites such as short-chain fatty acids and glutamatergic metabolites—which in turn plays a critical role in shaping neurodevelopmental trajectories via mechanisms including neurotransmitter signaling, neuroimmune interactions, and brain metabolic programming [17,18,19].

Among the studies addressing the topic of diet during pregnancy and childhood neurodevelopment, none have been looking specifically at sex-related differences. Cendra-Duarte and colleagues [20] explored this phenomenon but only related to glycemic index and glycemic load. They found that female offspring may be more vulnerable than males to behavioral disturbances linked to elevated maternal glycemic load during the first trimester of gestation. To our knowledge, no other study has examined sex-related differences in the impact of specific maternal dietary patterns on internalizing and externalizing behaviors, ADHD, and cognitive outcomes. Evidence suggests that fetal sex may modulate vulnerability to environmental stimuli, including maternal nutrition. Studies in animal models have shown that males and females exhibit different brain gene expression profiles in response to maternal diet, particularly in the context of obesity or high-fat or high-glycemic diets [21,22,23]. In light of these findings, we believe it is essential to analyze the associations between maternal diet and neurobehavioral development while taking the child’s sex into account, in order to identify potential sex-specific risk trajectories.

The aim of this study was to assess if a stricter adherence to specific dietary patterns during pregnancy affects behavioral (CBCL 1.5–5, Child Behavior Checklist) and cognitive (WPPSI-III, Wechsler Preschool and Primary Scale of Intelligence) outcomes at 4 years of age, and to explore potential sex-related differences in children within the Piccolipiù birth cohort, a large multicenter prospective study based in Italy [24]. Understanding these associations and the sex-related differences, if any, may contribute to early preventive strategies targeting maternal nutrition to promote optimal, maybe sex-specific, child development.

## 2. Materials and Methods

### 2.1. Study Population

This study is based on the Piccolipiù birth cohort, a multicenter Italian cohort designed to investigate the effects of prenatal and postnatal exposures on child health [24]. The cohort includes 3358 newborns enrolled at birth between 2011 and 2015 across selected hospitals in five Italian centers: Turin, Trieste, Florence, Viareggio, and Rome. Eligible participants were newborns born to mothers aged 18 years or older, residing in one of the participating centers and scheduled to give birth in these hospitals. At enrollment, data were collected through questionnaires and medical records, covering information on the newborns and their parents, including sociodemographic factors, lifestyle, occupational exposures, environmental conditions, and medical history. Follow-up assessments were conducted at 6, 12, 24, and 48 months using parental questionnaires. These follow-ups collected data on the child’s growth, living environment, health conditions, dietary habits, and any changes from baseline information. At 4 years of age, the follow-up focused on obesity, nutrition, and physical activity, alongside psychomotor and cognitive development assessments conducted by trained professionals. All mothers provided written informed consent at enrollment and at each subsequent follow-up. The Piccolipiù study was approved by the Ethics Committee of the Local Health Unit Roma E, national coordinator of the project (Prot. CE/82 09/06/2011), and of each local center [25].

### 2.2. Dietary Assessment

A structured questionnaire administered around the time of birth assessed maternal diet during pregnancy; the questionnaire also collected extensive information on demographics, education level, occupation, home environment, leisure activities, lifestyle, parental health, maternal diseases, and medication use during pregnancy and delivery. The section of the questionnaire focusing on maternal diet gathered data on the frequency of consumption of various food groups during pregnancy. Dietary information was collected using a structured food questionnaire. Although the questionnaire has not been formally validated, it was included as part of the multipurpose design of this study to capture general dietary patterns rather than to provide precise quantitative intake estimates. Participants reported how often they consumed different food items, using predefined frequency categories (e.g., never, less than once per week, 1–2 times per week, 3–5 times per week, 6–7 times per week, more than once per day). For caffeinated coffee and tea, and cola, participants indicated whether they consumed these beverages during pregnancy and, if so, the average number of cups per week in each trimester. The reference period was the whole pregnancy.

### 2.3. Neurodevelopmental and Cognitive Measurements

#### 2.3.1. Behavioral Assessment

Behavioral and emotional development at 4 years old was assessed using the Child Behavior Checklist for ages 1.5 to 5 years (CBCL 1.5–5, [26]). CBCL 1.5–5 is a psychological assessment instrument designed to collect information about the behaviors and social skills of children. The CBCL 1.5–5 is structured in the form of a questionnaire, completed by parents or guardians, who answer a series of questions that cover a broad spectrum of behaviors, both problematic and adaptive. It provides scores on seven syndrome scales (Emotionally Reactive, Anxious/Depressed, Somatic Complaints, Withdrawn, Sleep Problems, Attention Problems, Aggressive Behavior) and five DSM-oriented scales (Affective Problems, Anxiety Problems, Pervasive Developmental Problems, Attention Deficit Hyperactivity Disorder (ADHD), Oppositional Defiant Problems). Composite scores are generated for Internalizing Problems (summarizing Emotionally Reactive, Anxious/Depressed, Somatic Complaints, Withdrawn), Externalizing Problems (summarizing Attention Problems, Aggressive Behavior) and Total Problems (summarizing all the syndrome scales). In this study, we focused on three main outcomes: ADHD Problems, and Externalizing and Internalizing Problems. For CBCL scoring we have used percentiles (from 0 to 100), where 0 indicates the absence of behavioral or emotional problems, and 100 represents the highest severity. These percentile scores were used as continuous variables in the analyses. For analyses of clinical risk, raw CBCL scores were converted into T-scores using age- and sex-specific normative data derived from large population-based samples [26]. Participants were then grouped according to clinical thresholds: for ADHD, T-scores ≥65 and <70 were considered borderline, and ≥70 clinical; for internalizing and externalizing problems, scores ≥60 and ≥65 defined the borderline and clinical ranges, respectively [26]. Borderline and clinical scores were combined into a single outcome category.

#### 2.3.2. Cognitive Assessment

Cognitive development at 4 years of age was assessed using the Wechsler Preschool and Primary Scale of Intelligence—third edition (WPPSI-III) [27]. WPPSI-III is an individually administered psychometric instrument designed to assess cognitive development in preschool children, with an age range between 2 years and 6 months and 7 years and 3 months. The WPPSI-III scale is divided into two batteries of subtests, organized according to age groups (for our analysis the 4–7.25 years was used). Each battery of subtests allows the calculation of several main indices, namely, Verbal Intelligence Quotient (VIQ), Performance Intelligence Quotient (PIQ), and Total Intelligence Quotient (TIQ)); and of secondary indices, including Processing Speed Quotient (PSQ); and of optional indices, for example, General Language (GL) score. These subtests have a scoring range from 45 to 155, with higher scores indicating higher cognitive functioning.

### 2.4. Covariates

To investigate the association between maternal diet quality during pregnancy and children’s neurocognitive outcomes at 4 years of age, we accounted for several parental and child characteristics as potential confounders. These variables were selected based on their relevance to the exposure/outcome relationship and included maternal characteristics (age at delivery, pre-pregnancy BMI—body mass index, parity), sociodemographic factors (education, cohabiting and employment status, Equivalized Household Income Indicator (EHII) [28], center), maternal lifestyle factors (smoking before and during pregnancy, passive smoking during pregnancy, alcohol intake before and during pregnancy), neonatal characteristics (sex), and environmental factors (season of conception). Maternal and paternal education levels were categorized as low (no education, primary school, lower secondary school), medium (secondary high school), and high (post-secondary education or university degree). Maternal and paternal employment were coded as “always worked” (employed both before and after childbirth) or “not continuous” (employed only before or after). Cohabiting status was assessed at multiple time points (baseline, 12, 24, 48 months), and mothers were classified as “at least some time alone” if they lived alone with the child during any of these assessments. Socioeconomic status was estimated using the Equivalized Household Income Indicator (EHII) to estimate disposable monthly household income, standardized for household size and composition, and harmonized across European birth cohorts using EU-SILC reference data [28]. Income was categorized as low/medium or high based on a country-specific cut-off corresponding to the upper tertile of the 2011 EU-SILC reference distribution (households with at least one child ≤ 16 years). For our study population, this cut-off was EUR 1572.6, corresponding to a log-income value of 7.36. Participants with predicted log-EHII values < 7.36 were classified as low/medium income, and those with values ≥ 7.36 as high income.

Additional variables (e.g., breastfeeding in the first 6 months, gestational age (weeks), birth weight (grams), daycare within 24 months, exposure to smoke within 48 months) for children and mothers (e.g., pregnancy complication and maternal stress in the first 24 months after delivery) were collected for descriptive purposes only and were not included in the statistical analyses. Pregnancy complications included gestational diabetes, preeclampsia, hypertension, and thyroid diseases, and participants were classified as ‘Yes’ if they experienced at least one of these conditions during pregnancy. Maternal stress at 24 months was measured using the GHQ-12 (General Health Questionnaire, version at 12-item) and dichotomized as low (score 0–1) or moderate-to-high (≥2).

All data were collected through questionnaires administered at baseline and at 12, 24, and 48 months after delivery.

### 2.5. Statistical Analyses

#### 2.5.1. Descriptive Statistics

Characteristics of the study sample were described using mean values and standard deviations for continuous variables and frequencies, and percentages for categorical variables. For caffeinated coffee and tea, and cola, mean intake during pregnancy was estimated as the mean of the values for each trimester.

#### 2.5.2. Principal Component Analysis (PCA) for Dietary Pattern Identification

To identify specific dietary patterns, we applied Principal Component Analysis (prcomp function, “stats” package Rstudio, RStudio Team, 2022 [29]) to the standardized (mean = 0, SD = 1) food frequency variables from 3236 mothers who completed the pregnancy nutrition section of the baseline questionnaire. Cases with missing dietary data (*n* = 104; ~3% of the sample) were excluded from the analyses. The number of components retained was determined based on eigenvalues ≥2 (chosen to ensure robust factor structure), scree plot (elbow method), and interpretability of the extracted components. Varimax rotation (principal function, “psych” package Rstudio, RStudio Team, 2022 [30]) was applied to improve the interpretability of the components, assuming that the dietary patterns were orthogonal and independent from each other. A factor loading matrix was generated to extract weights (factor loading) for each food group, indicating the strength and direction of association between food group intake and dietary patterns. Food groups with factor loadings ≥0.40 were considered significant contributors to dietary patterns. Participants received an adherence score for each of the two identified dietary patterns. These scores were calculated by multiplying the matrix of standardized dietary intake data (Z) by the matrix of rotated factor loadings (L), according to the following formula: S = Z × L, where S is the matrix of adherence scores, reflecting how closely each participant’s diet aligned with the “Processed and high-fat foods” (RC1) and “Fresh foods and fish” (RC2) patterns.

#### 2.5.3. Regression Analyses

For each dietary pattern identified by PCA, we performed linear and logistic regression models. Linear regression models (lm function, “stats” package Rstudio, RStudio Team, 2022 [29]) were used to examine the association between maternal dietary pattern adherence scores (continuous variable) and child neurocognitive and behavioral outcomes (continuous scores). Logistic regression models (glm function, “stats” package Rstudio, RStudio Team, 2022 [29]) were used to assess the association between maternal dietary pattern and behavioral outcomes classified as being at clinical risk (i.e., borderline or clinical). For each regression two sets of multivariable models were fitted: i) including sex as a covariate; ii) sex-stratified analyses. Collinearity between covariates was assessed using Variance Inflation Factors (VIF), with all values below 1.5 indicating no concerning multicollinearity. Sensitivity analyses excluding certain maternal lifestyle variables (smoking or alcohol before pregnancy) yielded consistent estimates; thus, all covariates, as specified above, were retained as potential confounders. In all analyses, we used the CBCL and WPPSI scores as outcome variables. We have adopted a complete case approach (*n* = 2006).

All analyses were conducted using R statistical software (R Foundation for Statistical Computing, Vienna, Austria; version 4.2.2). We used RStudio (RStudio Team, 2022) as the integrated development environment. 

## 3. Results

### 3.1. General Characteristics

**Figure 1:**
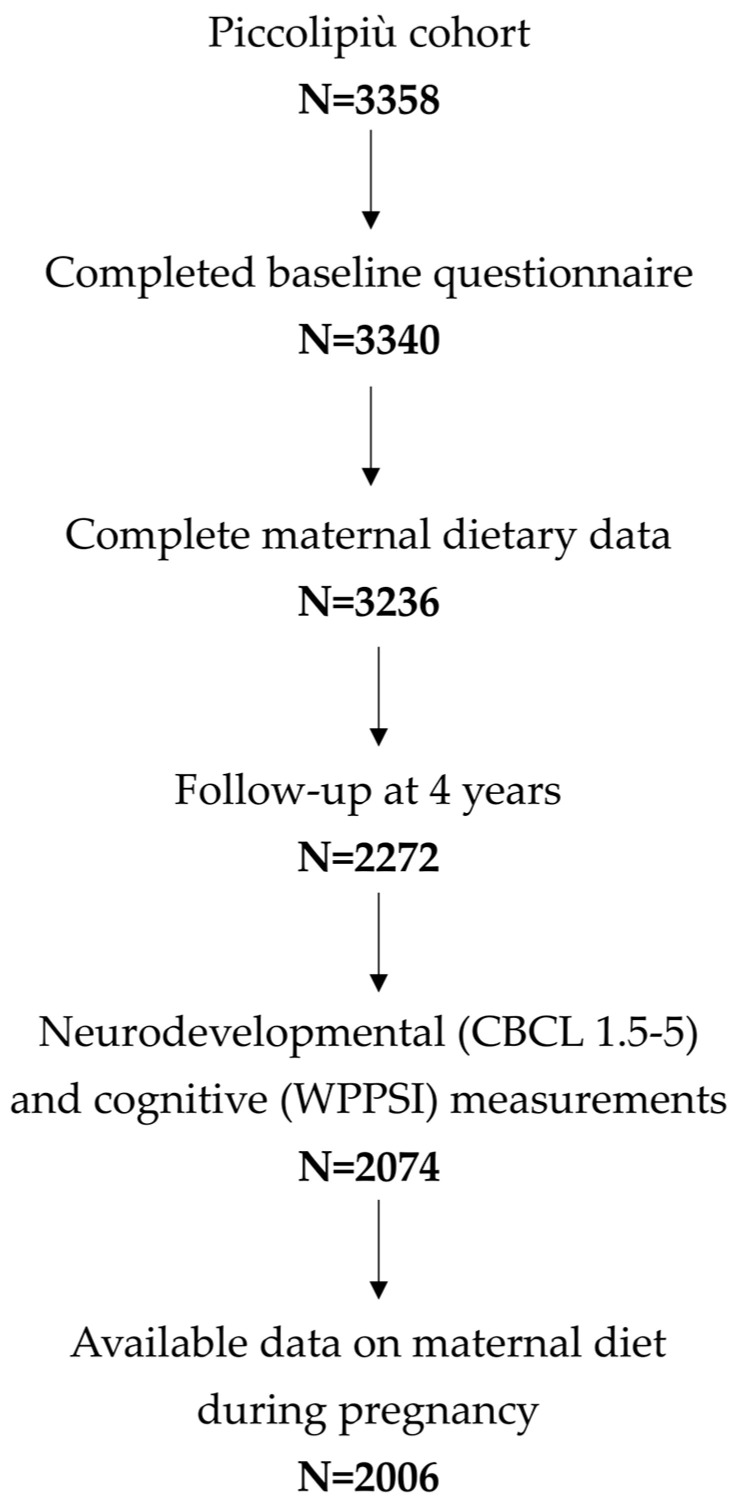
Flowchart illustrating the selection of the study cohort.

As shown in Figure 1, of the initial 3358 participants enrolled in the Piccolipiù cohort, 2006 mother/child pairs with complete data on maternal diet during pregnancy and neurodevelopmental outcomes at 4 years were included in the final analytical sample (60%).

The characteristics of participants are shown in Table 1A (parental characteristics) and Table 1B (children’s characteristics). The mean age at delivery was 34 years and 49% (*n* = 988) of the mothers had a high level of education (university or higher). As for socioeconomic status (EHII), we have an underrepresentation of the low/medium stratum of the income indicator, in agreement with the high education level. About half of the mothers (*n* = 1105) were employed before childbirth and returned to work within the first 4 years of the child’s life, and approximately 8% (*n* = 169) spent at least some time as single mothers during that period. Over 70% of mothers did not smoke (*n* = 1591) or suffer complications (*n* = 1663) during pregnancy and at least 50% (*n* = 1036) avoided alcohol consumption during pregnancy. Less than half of the children (*n* = 904) were exposed to secondhand parental smoke during the first 48 months of life, and 57% (*n* = 1135) attended daycare during the first 2 years of life. The mean birth weight was 3337 g, and the mean gestational age was 40 weeks.

Table 2 shows the frequency distribution of weekly consumption of the main food groups during pregnancy by frequency category (from never to more than once per day). For beverages (tea, coffee, and cola), the table reports the mean weekly consumption across the entire pregnancy, along with standard deviation, minimum, and maximum values. Only a few foods were consumed more than once a day by a significant proportion of participants: the highest rates were observed for fruit (35%, *n* = 692), pasta (32%, *n* = 647), and milk (14%, *n* = 287). Over 50% of participants reported eating raw or cooked vegetables at least three times a week. In contrast, less healthy or processed foods—such as fried foods, snacks, mayonnaise, and canned foods—were mostly consumed less than once a week or never by most participants (with percentages ranging from 67% to 88%). Regarding beverages, coffee was the most consumed, with an average of 3.8 servings per week.

The distribution of CBCL percentiles and WPPSI scores is presented in Table 3. The CBCL scores indicate that the cohort is generally composed of healthy children, with all mean scores falling below the 50th percentile. The relatively narrow distribution of WPPSI scores suggests that, in this cohort, intelligence levels were mostly within a typical range, with few extreme values.

**Figure 2:**
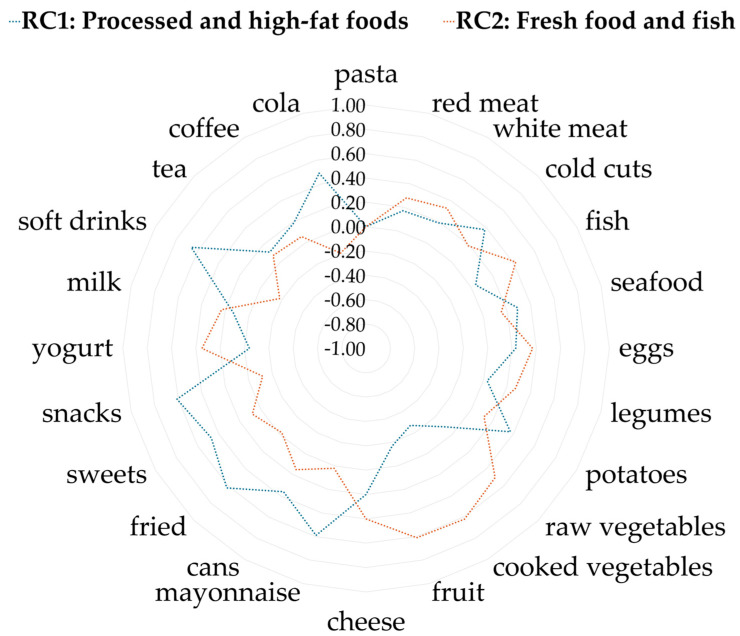
Radar plot of loadings obtained by Principal Component Analysis with varimax rotation. The blue line represents the first rotated component (RC1), while the orange line represents the second (RC2). Loading values indicate the contribution of each food to the respective food pattern.

Table S1 and Figure 2 show the factor loading matrix within the two main dietary patterns identified. The first pattern, represented by the first rotated component (RC1), called “Processed and high-fat foods”, was characterized by positive loadings of soft drinks (0.66) and cola (0.49), fried foods (0.62), snacks (0.61), sweets (0.47), and mayonnaise (0.59). The second model, represented by the second rotated component (RC2), called “Fresh foods and fish”, showed strong positive loadings for fish (0.42), cooked and raw vegetables (0.62 and 0.50, respectively) and fruits (0.61). For details on the eigenvalues and explained variance used to retain the two components, see Supplementary Table S2.

In Figure S1 and Table S3, different food categories are represented in terms of their RC1 and RC2 loads. Unhealthy foods, such as snacks, sweets, and fried foods, cluster in the lower right quadrant of the plot, reflecting high loadings on RC1 and low loadings on RC2. In contrast, healthier foods, such as raw and cooked vegetables, are positioned in the upper left quadrant, with high loadings on RC2 and low on RC1. Some healthy foods also appear in the upper right quadrant, closer to the origin, indicating a positive but weaker association with RC2. This visualization supports the identification of two distinct maternal dietary patterns during pregnancy.

### 3.2. Associations Between Maternal Dietary Patterns and Behavioral and Cognitive Disorders in Children at 4 Years of Age

The associations between maternal adherence to each dietary pattern and children’s CBCL 1.5–5 and WPPSI scores, estimated by multivariable linear regression models, are presented in Table 4, showing that each one-unit increase in maternal adherence to the “Processed and high fat foods” pattern was associated with worse offspring scores for externalizing behaviors (β = 0.88; 95%CI 0.28–1.49; *p* value = 0.004). Stratifying the analysis by child sex, the results were stronger for females than males, but the confidence intervals were overlapping. Consistently, the coefficient for the “Fresh food and fish” dietary pattern (RC2) and externalizing behavior was found to be negative (β = −0.25; 95%CI: −0.97–0.47), slightly stronger for females (β =−0.38; 95%CI: −1.42–0.66), but the upper bound of the 95%CI was greater than 0. Furthermore, none of the WPPSI scores were associated with RC1 or RC2.

### 3.3. Associations Between Maternal Dietary Patterns and Clinical Risk of Behavioral Problems in Children at 4 Years of Age

**Figure 3:**
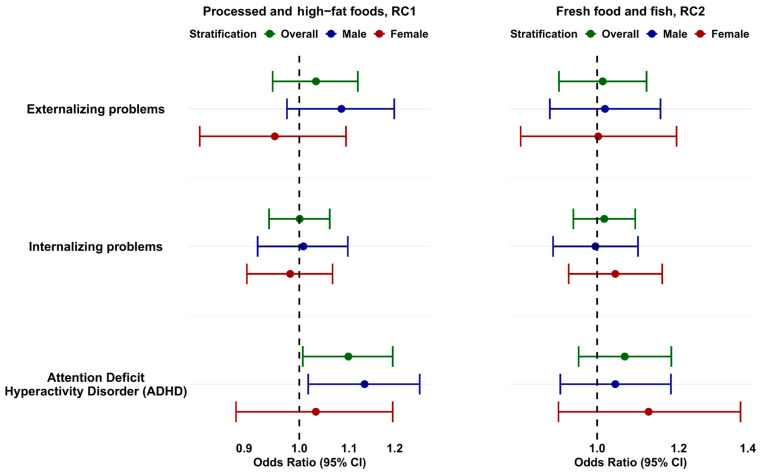
Odds Ratios (ORs) and 95% confidence intervals (CIs) for behavioral problems at 4 years of age (CBCL 1.5–5) in relation to maternal adherence to dietary patterns, based on the definition of children at risk, overall, and stratified by child sex. Multivariable logistic regression adjusted for maternal age, BMI, parity, maternal education, housing and employment status, Equivalized Household Income Indicator (EHII), center, smoking before and during pregnancy, passive smoking during pregnancy, alcohol intake before and during pregnancy, and season of conception. Adjustment for child sex is included only in models with all children. Dots represent Odds Ratios; whiskers indicate 95% confidence intervals.

As shown in Figure 3 and Table S4, maternal adherence to the “Processed and High-Fat Foods” (RC1) pattern was not associated with a clinical risk of externalizing or internalizing problems. However, children whose mothers showed high adherence to the RC1 pattern were more likely to have clinical scores for ADHD (OR (Odds Ratio) = 1.10; 95%CI: 1.01–1.20; *p* = 0.032). This indicates that for every one-unit increase in adherence to an unhealthy diet, the risk of ADHD increased significantly by 10%. When stratifying by sex, the association remained significant for males (OR = 1.13; 95%CI: 1.02–1.26; *p* = 0.021).

## 4. Discussion

This study evaluated the association between maternal dietary patterns during pregnancy and neurodevelopmental and behavioral outcomes in children at 4 years of age, using data from the Piccolipiù Italian birth cohort. A special focus has been put on sex-related differences. Two major dietary patterns were identified through PCA: one characterized by high consumption of processed and high-fat foods, and another by frequent intake of fresh foods and fish. Our findings suggest that higher adherence to a processed and high-fat diet during pregnancy is associated with increased behavioral problems. Specifically, children have a higher average score of externalizing problems if their mothers followed a diet with a higher fat intake (especially girls), while for males we found an increased clinical risk of ADHD within the same dietary pattern. These results point to possible sex-specific vulnerabilities. No association was found for cognitive outcomes.

The link between prenatal exposure to unhealthy diets and later behavioral problems is biologically plausible. Diets rich in sugars, saturated fats, and processed foods have been shown to induce systemic inflammation and alter the maternal gut microbiota, potentially influencing fetal brain development through neuroinflammatory pathways [31]. In contrast, diets rich in fruits, vegetables, and fish provide essential micronutrients and fatty acids that support neurogenesis and synaptogenesis [32,33]. While no significant associations were observed between the “Fresh foods and fish” pattern and behavioral or cognitive scores, the benefits of such a diet may manifest later or require greater variation in exposure to detect effects in relatively healthy cohorts.

This is one of the few studies that looked at differences in neurodevelopment between sexes. Understanding sex differences in such outcomes is of critical importance. In fact, it is known that many neurodevelopmental and psychiatric disorders exhibit significant differences in prevalence and presentation between sexes [34]. While genetics play a predominant role, with heritability up to 80% for ADHD and autism [16], environmental factors, including maternal diet, may interact with sex-specific pathways to influence neurodevelopment [8,9,10]. Maternal obesity and the consumption of a high-fat diet (HFD) have been consistently associated with adverse neurodevelopmental outcomes in children [35]. These include lower intelligence quotient (IQ) scores (2 to 3.4 points lower), poorer performance in reading and mathematics, and an increased risk of ADHD, autism spectrum disorder, developmental delay, and emotional/behavioral problems [35,36]. Mouse models of HFD-induced obesity show sex-specific behavioral outcomes: female offspring display decreased sociability, while male offspring exhibit hyperactivity in an open field test [35]. Data from the Boston Birth Cohort [37] show that low maternal HDL levels and suboptimal triglyceride levels are associated with an increased risk of ADHD, with this impact being “particularly pronounced in boys”. The risk of ADHD among boys was triple that of girls when mothers had low HDL levels. Our data confirm these results; we found that a diet richer in fat and processed food was associated with an increased risk of developing ADHD in males but not in females.

In general, sex-specific differences observed in our study reflect findings from other cohorts, where male and female fetuses respond differently to nutritional and hormonal environments in utero [38,39]. For instance, male offspring may be more vulnerable to early neurodevelopmental insults, while female offspring may display more externalizing behavior in response to later environmental exposures.

Possible explanation for sex-related differences included role of sex hormones [35], differential inflammatory and immune responses [35], epigenetic modifications and gene expression [22], and placenta/fetal interactions and metabolic demands [22].

It is also possible that some of the observed results are influenced by factors, such as the child’s nutrition (e.g., duration and quality of breastfeeding, introduction of solid foods) [40] and family lifestyle (e.g., level of physical activity, stress management, parenting practices) [41], which could be related to choices or habits already present during pregnancy, such as maternal dietary patterns.

As for cognition (WPPSI scores) we found no significant association with maternal dietary patterns in the whole sample and when stratifying by sex, although low variability in the score should be considered when interpreting these findings.

The strengths of this study include its prospective design, the use of validated instruments (WPPSI-III and CBCL), and adjustment for a comprehensive set of covariates. However, limitations must be acknowledged. Dietary data were based on self-reported intake, which may be subject to recall bias and social desirability bias. Moreover, the food questionnaire was not formally validated; however, as dietary intake was not the primary focus, it was used only to provide an approximate indication of dietary patterns rather than precise intake estimates. Furthermore, residual confounding cannot be ruled out, particularly related to unmeasured dietary exposures or parental characteristics. The generalizability of the findings may also be limited, as participants tended to be more educated and health-conscious compared to the general population.

## 5. Conclusions

Our findings suggest that maternal adherence to a processed and unhealthy dietary pattern during pregnancy is associated with an increased risk of behavioral problems, including externalizing behaviors and ADHD symptoms in children with some differences between sexes. Even though the results should be replicated in other studies, these associations highlight the potential importance of promoting healthy maternal diets during pregnancy as part of early prevention strategies aimed at supporting optimal neurodevelopment.

## Figures and Tables

**Table 1:** Study cohort characteristics, *n* = 2006.

**(A) Parental Characteristics**				
**Categorical Variables**	**Category**		***n* (%)**	
Maternal education at childbirth	Low		824 (41.08)	
	Medium		194 (9.67)	
	High		988 (49.25)	
	NA		-	
Paternal education at childbirth	Low		682 (34)	
	Medium		382 (19.04)	
	High		925 (46.11)	
	NA		17 (0.85)	
Equivalized Household Income Indicator (EHII)	Low/Medium		1131 (56.38)	
	High		770 (38.38)	
	NA		105 (5.23)	
Center	Florence		384 (19.14)	
	Viareggio		290 (14.46)	
	Rome		580 (28.91)	
	Turin		383 (19.09)	
	Trieste		369 (18.39)	
	NA		-	
Maternal employment (birth—48 months)	always worked		1105 (55.08)	
	not continuous		901 (44.92)	
	NA		-	
Paternal employment (birth—48 months)	always worked		1395 (69.54)	
	not continuous		611 (30.46)	
	NA		-	
Parity	Nulliparous		1182 (58.92)	
	Uniparous or multiparous		819 (40.83)	
	NA		-	
Cohabiting status in the first 48 months after delivery	at least some time alone		169 (8.42)	
	other cases		1430 (71.29)	
	NA		-	
Smoking before pregnancy	No		1043 (51.99)	
	Yes		957 (47.71)	
	NA		6 (0.30)	
Smoking in pregnancy	No		1591 (79.31)	
	Yes		414 (20.64)	
	NA		1 (0.05)	
Passive smoking in pregnancy	No		1345 (67.05)	
	Yes		510 (25.42)	
	NA		151 (7.53)	
Alcohol before pregnancy	No		765 (38.14)	
	Yes		1210 (60.32)	
	NA		31 (1.55)	
Alcohol in pregnancy	No		1036 (51.65)	
	Yes		963 (48.01)	
	NA		7 (0.35)	
Pregnancy complication	No		1663 (82.90)	
	Yes		328 (16.35)	
	NA		15 (0.75)	
Maternal stress in the first 24 months after delivery	Low		1250 (62.31)	
	Medium-High		620 (30.91)	
	NA		136 (6.78)	
**Continuous variables**			**Mean ± SD**	
Age at delivery (years)			33.9 ± 4.78	
Pre-pregnancy BMI (kg/m^2^)			22.5 ± 3.81	
**(B) Children’s Characteristics**				
**Categorical Variables**		**Category**		***n* (%)**
Exposure to smoke in the first 48 months		No		837 (41.72)
		Yes		904 (45.06)
		NA		265 (13.21)
Gender		Female		991 (49.40)
		Male		1015 (50.60)
		NA		-
Conception season		Autumn		399 (19.89)
		Spring		636 (31.70)
		Summer		517 (25.77)
		Winter		451 (22.48)
		NA		3 (0.15)
Breastfeeding in the first 6 months		No		110 (5.48)
		Yes		1873 (93.37)
		NA		23 (1.15)
Daycare in the first 24 months		No		689 (34.35)
		Yes		1135 (56.58)
		NA		182 (9.07)
**Continuous Variables**				**Mean ± SD**
Gestational age (weeks)				40 ± 1.32
Birth weight (grams)				3337.6 ± 442.29

The values are expressed as mean and standard deviation (SD) or frequency (*n*) and percentage (%), as appropriate. BMI: body mass index. NA: not available.

**Table 2:** Distribution (*n*, %) of weekly food intake frequencies during pregnancy and mean and SD weekly consumption of beverages: (**a**) values represent the number of participants reporting each frequency category (row percentages are shown in parentheses) for the listed food items; (**b**) beverage consumption (tea, coffee, cola) is expressed as the average number of servings per week across the three trimesters.

**(a)**								
**Food Item**	**Never**		**Less than Once/Week**	**1–2 Times/Week**	**3–5 Times/Week**		**6–7 Times/Week**	**More than Once/Day**
white meat	47 (2.3)		176 (8.8)	1012 (50.4)	669 (33.3)		95 (4.7)	7 (0.3)
soft drinks	493 (24.6)		791 (39.4)	455 (22.7)	170 (8.5)		54 (2.7)	43 (2.1)
cooked vegetables	25 (1.2)		121 (6)	530 (26.4)	794 (39.6)		355 (17.7)	181 (9)
raw vegetables	221 (11)		242 (12.1)	453 (22.6)	587 (29.3)		306 (15.3)	197 (9.8)
sweets	41 (2)		309 (15.4)	471 (23.5)	597 (29.8)		443 (22.1)	145 (7.2)
cheese	73 (3.6)		248 (12.4)	768 (38.3)	715 (35.6)		174 (8.7)	28 (1.4)
fried food	326 (16.3)		1338 (66.7)	293 (14.6)	41 (2)		6 (0.3)	2 (0.1)
fruit	15 (0.7)		55 (2.7)	182 (9.1)	488 (24.3)		574 (28.6)	692 (34.5)
seafood	1048 (52.2)		830 (41.4)	114 (5.7)	13 (0.6)		0 (0)	1 (0)
milk	201 (10)		143 (7.1)	141 (7)	274 (13.7)		960 (47.9)	287 (14.3)
legumes	62 (3.1)		637 (31.8)	998 (49.8)	256 (12.8)		41 (2)	12 (0.6)
mayonnaise	949 (47.3)		814 (40.6)	185 (9.2)	52 (2.6)		5 (0.2)	1 (0)
pasta	1 (0)		24 (1.2)	166 (8.3)	535 (26.7)		633 (31.6)	647 (32.3)
potatoes	28 (1.4)		623 (31.1)	1062 (52.9)	268 (13.4)		22 (1.1)	3 (0.1)
fish	77 (3.8)		510 (25.4)	1147 (57.2)	235 (11.7)		27 (1.3)	10 (0.5)
red meat	73 (3.6)		307 (15.3)	996 (49.7)	538 (26.8)		79 (3.9)	13 (0.6)
cold cuts	487 (24.3)		601 (30)	624 (31.1)	244 (12.2)		44 (2.2)	6 (0.3)
cans	730 (36.4)		809 (40.3)	370 (18.4)	87 (4.3)		9 (0.4)	1 (0)
snacks	428 (21.3)		924 (46.1)	437 (21.8)	165 (8.2)		37 (1.8)	15 (0.7)
eggs	98 (4.9)		845 (42.1)	973 (48.5)	81 (4)		7 (0.3)	2 (0.1)
yogurt	196 (9.8)		342 (17)	529 (26.4)	502 (25)		357 (17.8)	80 (4)
**(b)**								
**Beverage**		**Mean ± SD**				**Min-Max**		
tea		1.14 ± 1.95				0–20		
coffee		3.79 ± 4.65				0–70		
cola		1.24 ± 2.35				0–60		

**Table 3:** Score distribution in children at age 4 in CBCL and WPPSI subtests, for whom maternal dietary data during pregnancy are available, *n* = 2006.

Neurodevelopmental and Cognitive Measurements	Median (IQR)
**CBCL, *n* = 1995**	
Externalizing problems	46 (19–74)
Internalizing problems	45 (22–75)
Attention Deficit Hyperactivity Disorder (ADHD)	39 (17–61)
	**Mean ± SD**
**WPPSI, *n* = 1890**	
Verbal Intelligence Quotient (VIQ)	114 ± 12
Performance Intelligence Quotient (PIQ)	112 ± 14
General Language (GL)	109 ± 11
Processing Speed Quotient (PSQ)	103 ± 14
Total Intelligence Quotient (TIQ)	114 ± 13

CBCL 1.5–5: Child Behavior Checklist 1.5–5. WPPSI: Wechsler Preschool and Primary Scale of Intelligence.

**Table 4:** Beta coefficients from multivariable linear regression models for the association between maternal adherence to dietary patterns and behavioral and cognitive problems at 4 years of age, overall and stratified by child gender.

	Processed and High-Fat Foods (RC1)						Fresh Food and Fish (RC2)					
Neurodevelopmental and Cognitive Measurements	All	Females		Males			All		Females		Males	
**CBCL**	**β (95%CI)**	***p* Value**	**β (95%CI)**	***p* Value**	**β (95%CI)**	***p* Value**	**β (95%CI)**	***p* Value**	**β (95%CI)**	***p* Value**	**β (95%CI)**	***p* Value**
Externalizing problems	0.88 (0.28–1.49)	**0.004**	0.99 (0.17–1.81)	**0.017**	0.7 (−0.21–1.61)	0.133	−0.25 (−0.97–0.47)	0.497	−0.38 (−1.42–0.66)	0.471	−0.21 (−1.23–0.81)	0.686
Internalizing problems	0.26 (−0.34–0.87)	0.394	0.16 (−0.67–1.00)	0.705	0.28 (−0.62–1.18)	0.540	−0.03 (−0.76–0.69)	0.928	0.18 (−0.88–1.24)	0.741	−0.23 (−1.24–0.78)	0.654
Attention Deficit Hyperactivity Disorder (ADHD)	0.37 (−0.26–1.00)	0.254	0.01 (−0.85–0.87)	0.978	0.77 (−0.17–1.71)	0.110	0.05 (−0.70–0.81)	0.889	0.43 (−0.66–1.51)	0.442	−0.31 (−1.37–0.75)	0.565
**WPPSI**												
Verbal Intelligence Quotient (VIQ)	−0.09 (−0.32–0.13)	0.428	−0.17 (−0.45–0.11)	0.245	0.01 (−0.35–0.38)	0.943	0.03 (−0.24–0.30)	0.819	0.14 (−0.22–0.50)	0.457	−0.08 (−0.49–0.33)	0.693
Performance Intelligence Quotient (PIQ)	0.19 (−0.07–0.45)	0.161	0.20 (−0.14–0.54)	0.247	0.20 (−0.21–0.61)	0.346	0.14 (−0.18–0.45)	0.396	0.23 (−0.20–0.66)	0.289	0.02 (−0.44–0.49)	0.916
General Language (GL)	−0.16 (−0.41–0.08)	0.189	−0.26 (−0.56–0.03)	0.082	−0.04 (−0.45–0.36)	0.843	0.08 (−0.20–0.37)	0.577	0.19 (−0.20–0.57)	0.337	0.00 (−0.44–0.43)	0.990
Processing Speed Quotient (PSQ)	0.14 (−0.14–0.42)	0.331	0.13 (−0.26–0.52)	0.508	0.12 (−0.31–0.54)	0.589	−0.28 (−0.62–0.06)	0.101	−0.40 (−0.89–0.10)	0.116	−0.21 (−0.67–0.26)	0.391
Total Intelligence Quotient (TIQ)	0.09 (−0.16–0.34)	0.459	0.08 (−0.23–0.39)	0.608	0.12 (−0.29–0.52)	0.568	0.05 (−0.25–0.35)	0.744	0.17 (−0.23–0.56)	0.410	−0.07 (−0.52–0.38)	0.750

Models are adjusted for maternal age at delivery, BMI, parity, education, cohabiting and employment status, Equivalized Household Income Indicator (EHII), center, smoking before and during pregnancy, passive smoking during pregnancy, alcohol intake before and during pregnancy, and season of conception. Adjustment for child sex is included only in models with all children. The values of *n* indicate the number of observations included in each model. *p* values < 0.05 are highlighted in bold. CBCL 1.5–5: Child Behavior Checklist 1.5–5. WPPSI: Wechsler Preschool and Primary Scale of Intelligence. CI: confidence interval.

## Data Availability

The data presented in this study are available on reasonable request from the corresponding author and agreement on the terms of data use and publication of the results. All proposals requesting data access will need to specify how the data will be used.

## Supplementary Materials

The following supporting information can be downloaded at https://www.mdpi.com/article/10.3390/nu17172814/s1: Table S1: Factor loadings of individual food groups on the two principal components derived from the Principal Component Analysis; Table S2: Eigenvalues and explained variance from Principal Component Analysis; Figure S1: Distribution of food items in the loading space of the first two rotated components (RC1 and RC2), stratified by food group; Table S3: Nutritional classification of food items and interpretation of their distribution in the rotated component space; Table S4: Odds Ratios (ORs) and 95% confidence intervals (CIs) for behavioral problems at 4 years of age (CBCL 1.5–5) in relation to maternal dietary patterns, based on the definition of children at risk, overall and stratified by child sex.
