# Supplementary material for: Maternal Dietary Pattern in Pregnancy and Behavioral Outcomes at 4 Years of Age in the Piccolipiù Cohort: Potential Sex-Related Differences
Source: Nutrients. 2025 Aug 29;17(17):2814. doi: 10.3390/nu17172814 (PMC12430498; doi:10.3390/nu17172814)
Supplement: Supplementary file 1 [file nutrients-17-02814-s001.zip › nutrients-3819859-supplementary.pdf]

Table S1. Factor loadings of individual food groups on the two principal components derived from the Principal Component Analysis.

**Food Groups**	**Processed and high-fat foods, RC1**	**Fresh food and fish, RC2**
pasta	0.17	0.37
red meat	0.17	0.28
white meat	0.19	0.33
cold cuts	0.38	0.19
fish	0.04	**0.42**
seafood	0.29	0.15
eggs	0.23	0.37
legumes	0.03	0.27
potatoes	0.37	0.12
raw vegetables	-0.09	**0.5**
cooked vegetables	-0.27	**0.62**
fruit	-0.17	**0.61**
cheese	0.2	0.4
mayonnaise	**0.59**	0.02
cans	0.36	0.15
fried	**0.62**	-0.02
sweets	**0.47**	0.08
snacks	**0.61**	-0.12
yogurt	-0.04	0.35
milk	0.13	0.23
soft drinks	**0.66**	-0.18
tea	0.12	0.08
coffee	0.19	0.06
cola	**0.49**	-0.19

RC1: Rotated Component 1; RC2: Rotated Component 2. Loadings highlighted in bold indicate foods that are significant contributors to the dietary pattern.

Table S2. Eigenvalues and explained variance from Principal Component Analysis.

Component	Eigenvalue	Proportion of Variance Explained,%	Cumulative Proportion, %
PC1	2.8685	0.1195	0.1195
PC2	2.2389	0.0933	0.2128
PC3	1.5684	0.0654	0.2782
PC4	1.4548	0.0606	0.3388
PC5	1.2477	0.0520	0.3908
PC6	1.1961	0.0498	0.4406
PC7	1.0929	0.0455	0.4861
PC8	1.0281	0.0428	0.5290
PC9	0.9534	0.0397	0.5687
PC10	0.9304	0.0388	0.6075
PC11	0.8927	0.0372	0.6447
PC12	0.8195	0.0341	0.6788
PC13	0.7863	0.0328	0.7116
PC14	0.7830	0.0326	0.7442
PC15	0.7496	0.0312	0.7754
PC16	0.6939	0.0289	0.8043
PC17	0.6882	0.0287	0.8330
PC18	0.6445	0.0269	0.8599
PC19	0.6282	0.0262	0.8860
PC20	0.6103	0.0254	0.9115
PC21	0.5982	0.0249	0.9364
PC22	0.5711	0.0238	0.9602
PC23	0.5554	0.0231	0.9833
PC24	0.3999	0.0167	1.0000

The eigenvalues and the proportion of total variance explained by each of the 24 components derived from the principal component analysis (PCA) of dietary intake data. Components are listed in descending order of explained variance. Each eigenvalue represents the amount of variance in the original data explained by a given principal component.

Figure S1. Distribution of food items in the loading space of the first two rotated components (RC1 and RC2), stratified by food group.

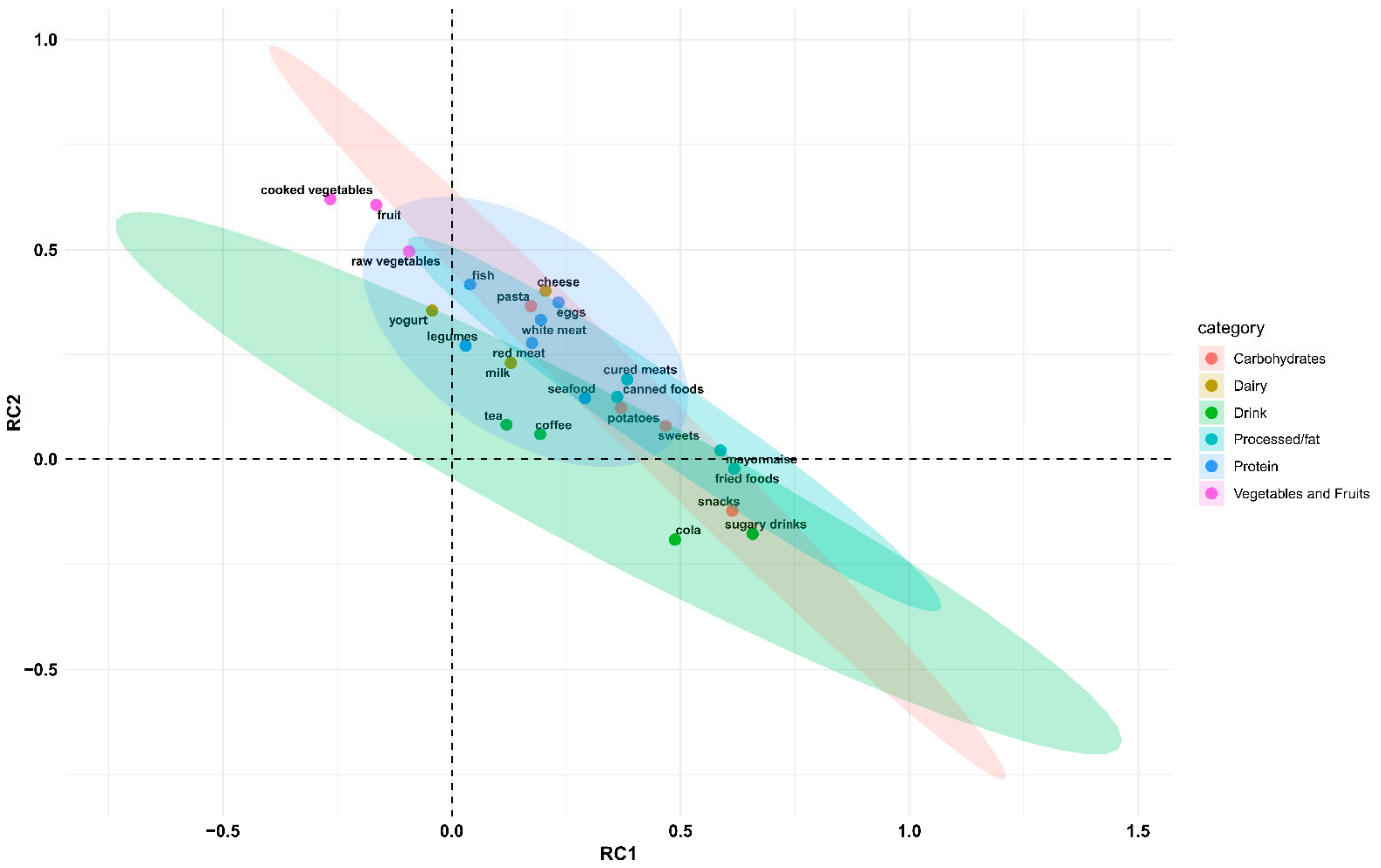

Colored ellipses represent the distribution of food items within each nutritional category in the rotated loading space. Each ellipse summarizes the central tendency and variability of items belonging to the same group. Ellipses are only shown for food categories with sufficient data points to estimate variance in both dimensions. RC1: Rotated Component 1; RC2: Rotated Component 2. Food groups were categorized according to their nutritional characteristics and their distribution within the rotated component space. The food groups are: Carbohydrates (pasta, potatoes, sweets, snacks), Dairy (yogurt, milk, cheese), Drinks (soft drinks, tea, coffee, cola), Processed/fat (mayonnaise, fried foods, canned foods, cured meats), Protein (red meat, white meat, fish, eggs, seafood, legumes), and Vegetables and fruits (cooked and raw vegetables, fruit).

Table S3. Nutritional classification of food items and interpretation of their distribution in the rotated component space.

FOOD GROUP	INCLUDES	BIPLOT MAIN POSITION	INTERPRETATION
Carbohydrates	pasta, potatoes, sweets, snacks	High on RC1, medium on RC2	associated with a pattern rich in energy and sugars
Dairy	yogurt, milk, cheese	Center	neutral, not strongly characterizing
Drink	soft drinks, tea, coffee, cola	Right, low on RC2	very processed, not very healthy
Processed /fat	mayonnaise, fried foods, canned foods, cured meats	High on RC1, low on RC2	typical of the processed pattern
Protein	red meat, white meat, fish, eggs, seafood, legumes	Negative on RC1, medium-high on RC2	associated with the healthy pattern
Vegetables and fruit	cooked/raw vegetables, fruit	Low on RC1, high on RC2	strong characterization of the fresh/healthy pattern

Each food item was assigned to a broader food group based on its nutritional characteristics. The table reports the main biplot positioning of each group according to their loadings on the first two rotated components (RC1 and RC2), and their interpretation in terms of dietary pattern.

Table S4. Odds ratios (ORs) and 95% confidence intervals (CIs) for behavioral problems at 4 years of age (CBCL 1.5–5) in relation to maternal dietary patterns, based on the definition of children at risk, overall and stratified by child sex.

	Processed and High fat foods (RC1)						Fresh food and Fish (RC2)					
**Neurodevelopmental Measurements**	**All**		**Females**		**Males**		**All**		**Females**		**Males**	
**CBCL**	**OR (95%CI)**	**p value**	**OR (95%CI)**	**p value**	**OR (95%CI)**	**p value**	**OR (95%CI)**	**p value**	**OR (95%CI)**	**p value**	**OR (95%CI)**	**p value**
Externalizing problems	1.03(0.95-1.12)	0.442	0.95(0.83-1.09)	0.510	1.08(0.98-1.2)	0.122	1.01(0.92-1.12)	0.805	1.00(0.84-1.19)	0.978	1.02(0.9-1.15)	0.781
Internalizing problems	1.00(0.94-1.06)	0.978	0.98(0.9-1.07)	0.676	1.01(0.92-1.1)	0.857	1.02(0.95-1.09)	0.653	1.04(0.94-1.16)	0.447	1.00(0.91-1.1)	0.937
Attention Deficit Hyperactivity Disorder (ADHD)	1.10(1.01-1.20)	**0.032**	1.03(0.89-1.20)	0.677	1.13(1.02-1.26)	**0.021**	1.06(0.96-1.18)	0.243	1.12(0.92-1.38)	0.267	1.04(0.92-1.18)	0.519

Multivariable logistic regression adjusted for maternal age, BMI, parity, maternal education, housing and employment status, socio-economic indicator (Pizzi et al., 2020), study center, smoking before and during pregnancy, passive smoking during pregnancy, alcohol intake before and during pregnancy, exposure to smoking within 48 months, nursery attendance within 24 months, and season of conception. Adjustment for child sex is included only in models with all children. P-values < 0.05 are highlighted in bold. CBCL 1.5-5: Child Behavior Checklist 1.5-5. CI: confidence interval.
